# *Zingiber officinale *acts as a nutraceutical agent against liver fibrosis

**DOI:** 10.1186/1743-7075-8-40

**Published:** 2011-06-20

**Authors:** Tarek K Motawi, Manal A Hamed, Manal H Shabana, Reem M Hashem, Asmaa F Aboul Naser

**Affiliations:** 1 Biochemistry Department, Faculty of Pharmacy, Cairo University, Kasr El-Aini St., Cairo, 11562, Egypt; 2 Therapeutic Chemistry Department, National Research Center, El-Tahrir St., Dokki, Cairo, 12311, Egypt; 3 Phytochemistry and Plant Systematic Department, National Research Center, El-Tahrir St., Dokki, Cairo, 12311, Egypt; 4 Biochemistry Department, Faculty of Pharmacy, Beni-Seuf University, Salah Salem St., Beni-Seuf, 62511, Egypt

**Keywords:** *Zingiber officinale*, liver fibrosis, enzymes, antioxidants, histology

## Abstract

**Background/objective:**

*Zingiber officinale *Roscoe (ginger) (*Zingiberaceae*) has been cultivated for thousands of years both as a spice and for medicinal purposes. Ginger rhizomes successive extracts (petroleum ether, chloroform and ethanol) were examined against liver fibrosis induced by carbon tetrachloride in rats.

**Results:**

The evaluation was done through measuring antioxidant parameters; glutathione (GSH), total superoxide dismutase (SOD) and malondialdehyde (MDA). Liver marker enzymes; succinate and lactate dehydrogenases (SDH and LDH), glucose-6-phosphatase (G-6-Pase), acid phosphatase (AP), 5'- nucleotidase (5'NT) and liver function enzymes; aspartate and alanine aminotransferases (AST and ALT) as well as cholestatic markers; alkaline phosphatase (ALP), gamma glutamyl transferase (GGT), total bilirubin were estimated. Liver histopathological analysis and collagen content were also evaluated. Treatments with the selected extracts significantly increased GSH, SOD, SDH, LDH, G-6-Pase, AP and 5'NT. However, MDA, AST, ALT ALP, GGT and total bilirubin were significantly decreased.

**Conclusions:**

Extracts of ginger, particularly the ethanol one resulted in an attractive candidate for the treatment of liver fibrosis induced by CCl_4_. Further studies are required in order to identify the molecules responsible of the pharmacological activity.

## 1. Background

Liver plays a pivotal role in regulating various physiological processes in the body such as metabolism, secretion and storage. It has great capacity to detoxicate toxic substances and synthesize useful principles. Therefore, damage on the liver inflicted by hepatotoxic agents is of grave consequences [1].

Evidences developed over the last years have suggested that various forms of liver injuries may be caused by free radical formation and subsequent oxidative stress. It is believed that reactive oxygen species (ROS), such as hydroxyl radical, superoxide radical anion and nitric oxide may injure cell membranes through lipid peroxidation [2]. Apparently ROS modify or damage biomolecules, i.e., proteins, lipids, carbohydrates and DNA [3]. Oxidative stress lead to the formation of glycoxidation products, including advanced glycation end products (AGEs - among them *Nε*-(carboxymethyl) lysine (CML) is best known), and advanced oxidation protein products (AOPPs). The receptor for advanced glycation end products (RAGE) is a signal transduction receptor that binds both AGEs and AOPPs. RAGE is expressed by hepatic stellate cells and myofibroblasts, which are the relevant cells for fibrogenesis of chronic liver disease. Both AGEs and AOPPs trigger the inflammatory response *via *interaction with RAGE and by causing activation of nuclear factor NF-κB [4]. Since advanced oxidation protein products are not only markers of oxidative stress but also act as inflammatory mediators [5], the knowledge of AOPPs pathophysiology in chronic liver disease could provide valuable information with respect to the relationship between oxidative stress and the inflammatory response related to liver fibrosis [6].

Carbon tetrachloride (CCl_4_), as a xenobiotic, caused oxidative stress and may injuries hepatic cells [7]. Many studies have established the fact that CCl_4 _is metabolized in the liver into a highly reactive substance, trichloromethyl, which initiate free radicals that mediate lipid peroxidation [8].

For this reason, anti-oxidation is an extremely significant activity which can be used as a preventive agent against diseases [9]. In spite of tremendous advances in modern medicine, there are no effective drugs available to improve liver function, offer protection or help to regenerate hepatic cells [1]. In absence of reliable liver-protective drugs, therefore, attention is focused on natural antioxidants. Natural antioxidants are polyphenol compounds which are found in all plants [10].

Ginger (*Zingiber officinale *Roscoe; Zingiberaceae) has been used as a spice for over thousand years [11]. Its roots contain polyphenol compounds (6-gingerol and shogaols), which have a high antioxidant activity [12]. In addition, ginger reported as detoxifying agent against alcohol abuse [13] and bromobenzene intoxication [14]. Matsuda et al. [15] and Habib et al. [16] mentioned its antidiabetic, antihyperlipidimic and hepatic anticancer effect.

According to glossary produced by American Diabetics and Association, neutriceutics are substances considered as food or a part of it that offers health or medicinal benefit, including prevention and treatment of diseases [17]. Some of the natural products find their use not as pharmaceuticals (real medicine) but as a novel class of dietary supplements or nutraceuticals that fall well into the concept of functional foods. Ginger has been listed in ''Generally Recognized as Safe'' (GRAS) document of the US FDA, where a dose of 0.5-1.0 g of ginger powder ingested 2-3 times for periods ranging from 3 months to 2.5 years did not cause any adverse effects [18].

Despite the favorable ethnopharmacological properties of ginger, its protective effect against liver fibrosis has not previously been explored and its role as diminished factor of fibrosis could be a marker of therapeutic benefit. The aim of the present study was to evaluate successive ginger extracts (petroleum ether, chloroform and ethanol) against liver fibrosis induced by CCl_4 _in rats. The evaluation was done through measuring certain antioxidant parameters, hepatic marker enzymes, liver function indices, cholestatic biomarkers and histological analysis of the liver sections.

## 2. Material and methods

### 2.1. Chemicals

All chemicals in the present study were of analytical grade, product of Sigma (US), Merck (Germany) and BDH (England).

### 2.2. Plant collection

Ginger rhizomes were purchased from local market (Hyper One, 6^th ^October City, Egypt) and identified by Dr. Manal Shabana, Phytochemistry and Plant Systematic Department, National Research Center, Egypt. Voucher specimen (ZOR-2010) was deposited as a reference at Therapeutic Chemistry Department, NRC. Dried rhizomes were ground in a grinder with 2 mm diameter mesh and kept in tightly closed container until needed.

### 2.3. Plant Extraction

The dried powdered rhizomes (500 g) were successively extracted in a Soxhlet apparatus using solvents of increasing polarities: petroleum ether (40-60°C), chloroform and 95% ethanol for 72 h of each solvent [19]. Solvent removal was carried by evaporation under reduced pressure at 40°C yielding semisolid residues of 1.3, 0.80 and 2.35% w/w, respectively.

### 2.4. Phytochemical screening

All extracts were tested for carbohydrates, sterols, terpenes [20], flavonoids, tannins, oils [21] and alkaloids [22].

### 2.5. Animals

Male Wistar albino rats (100: 120 g) were selected for this study. They were obtained from the Animal House, National Research Center, Egypt. All animals were kept in controlled environment of air and temperature with access of water and diet. Anesthetic procedures and handling with animals complied with the ethical guidelines of Medical Ethical Committee of National Research Centre in Egypt (Approval no: 10031).

### 2.6. Doses and route of administration

Administration regimen was twice a week for six consecutive weeks. CCl_4 _(0.5 ml/kg) was suspended in olive oil (1:9 v/v) and injected intraperitoneally [23]. Ginger extracts were administrated orally at a dose 200 mg/kg) [14]. Silymarin; a reference herbal drug was orally administered at a dose 100 mg/kg [24].

Normal control group was orally vehicle with 0.5 ml normal physiological saline and intraperitoneally received 0.5 ml olive oil. Control groups treated with ginger extracts were intraperitoneally vehicle with 0.5 ml olive oil. CCl_4 _group was orally vehicle with 0.5 ml of normal physiological saline.

### 2.7. Experimental groups

72 male Wistar strain albino rats were used in this study. Animals were divided into 9 groups (8 rats each). Group1 served as normal healthy control rats. Groups 2-4 were normal healthy rats administered with ginger extracts (petroleum ether, chloroform and ethanol). Group 5 injected with CCl_4_. Groups 6-8 forced at the same time with CCl_4 _and ginger extracts. Group 9 forced with CCl_4 _and silymarin drug.

### 2.8. Sample preparations

Serum sample: Blood collected from each animal by puncture the sublingual vein in clean and dry test tube, left 10 min to clot and centrifuged at 3000 rpm (4°C) for serum separation. The separated serum was stored at -80°C for further determinations of liver function enzymes, cholestatic biomarkers and serum protein.

Liver tissue was homogenized in normal physiological saline solution (0.9% NaCl) (1:9 w/v). The homogenate was centrifuged at 4°C for 5 min at 3000 rpm. The supernatant was used for estimation of liver marker enzymes and the antioxidant parameters.

### 2.9. Biochemical assays

#### 2.9.1. Antioxidant parameters

Malondialdehyde as a product of polyunsaturated fatty acids oxidation was determined by the method of Buege and Aust [25]. Its concentration was calculated using the extinction coefficient value 1.56 × 10^5 ^M^-1 ^cm^-1 ^and read at 535 nm. Glutathione was assayed by the method of Moron et al. [26] using dithiobis-2-nitrobenzoic acid (DTNB) in PBS. The reaction colour was read at 412 nm. Total superoxide dismutase was estimated by method of Nishikimi et al. [27], where the increase in NADH oxidation was measured at 560 nm using its molar extinction coefficient 6.22 × 10^3 ^M^-1 ^cm^-1^.

#### 2.9.2. Hepatic cell organelles markers

Succinate dehydrogenase; a marker for mitochondria was estimated by the method of Rice and Shelton [28], where the reduction of flavin adenine dinucleotide was coupled with a reduction of tetrazolium salt as 2-p-iodophenyl-3-p-nitrophenyl-5-phenyl tetrazolium chloride (INT) and the produced formazan of INT was measured colorimetrically at 490 nm. Lactate dehydrogenase; a marker for cytoplasm was estimated by the method of Babson and Babson [29], where the reduction of nucleoside derived amino acids (NAD) was coupled with the reduction of tetrazolium salt and the produced formazan of INT was measured colorimetrically at 503 nm. The three enzymes, G-6-Pase (microsome marker), acid phosphatase (lysosome marker) and 5'- nucleotidase (plasma membrane marker) were measured colorimetrically as inorganic phosphorus released at 660 nm [30-32], respectively.

#### 2.9.3. Serum biomarkers for liver function tests and total protein level

Aspartate and alanine amintransferases were measured by the method of Gella et al. [33], where the transfer of amino group from aspartate or alanine formed oxalacetate or pyruvate, respectively and the developed colour was measured at 520 nm. Total protein was assayed by the method of Bradford [34], where Coomassie Brilliant Blue dye reacted with Bradford reagent and gave a blue complex at 595 nm.

#### 2.9.4. Cholestatic indices

Alkaline phosphatase catalyzed in alkaline medium the transfer of phosphate group from 4-nitrophosphatase to 2-amino-2-methyl-1-propanol (AMP) and librated 4-nitrophenol. The developed colour was measured at 510 nm [35]. GGT was estimated by the method of Szasz [36], where GGT enzyme reacted with L-γ - glutamyl-3-carboxy-p-nitroanilide and glycyl-glycine to give L-γ - glutamyl-glycyl-glycine and 5-amino-2-nitrobenzoate. The decrease in absorbance was read at 450 nm at 1 min intervals for 3 minutes. Total bilirubin was measured by the method of Doumas et al. [37], where it reacted with diazotized sulfanilic acid in the presence of caffeine with final azo-pigment product. The developed colour was read at 546 nm.

### 2.10. Histopathological study

Liver slices were fixed in 10% paraformaldehyde and embedded in paraffin wax blocks. Sections of 5 μm thick were stained with hematoxylin & eosin (H&E) and Masson's trichrome, then examined under light microscope for determination of pathological changes [38]. Collagen content was determined in Masson's trichrome sections and expressed as collagen volume, where volume of collagen (%) = number of points failing on 10 successive fields/number of points in the reticule (1 cm^2 ^eye piece reticule) × 100 [39].

### 2.11. Statistical analysis and calculations

All data were expressed as mean ± SD of eight rats in each group. Statistical analysis was carried out by one-way analysis of variance (ANOVA), Costat Software Computer Program. Significance difference between groups was at p < 0.05.

% change = control mean - treated mean/control mean × 100.

% improvement = treated mean - injured mean/control mean × 100.

## 3. Results

### 3.1. Phytochemical screening

Phytochemical screening of ginger ethanolic extract revealed abundant presence of flavonoids and tannins. High concentration of carbohydrates and moderate concentration of alkaloids were recorded. Chloroformic extract contained moderate concentrations of sterols and terpenes, while petroleum ether one contained moderate lipids content (Table 1).

**Table 1:** Phytochemical screening of successive ginger extracts

Constituents	Petroleum ether	Chloroform	Ethanol
Carbohydrate	-	-	+++
Flavonoids	-	-	++++
Tannins	-	-	++++
Sterols	-	++	-
Terpenes	-	++	-
Alkaloids	-	-	-
Oils	++	-	-

●^- ^= Not present.

● + = Present in low concentration.

● ++ = Present in moderately high concentration.

● +++ = Present in very high concentration.

● ++++ = Abundantly present.

### 3.2. Extracts safety

Normal healthy rats administrated with ethanol and chloroform extracts recorded insignificant changes in all parameters under investigation revealing extracts safety. Petroleum ether extract showed significant changes in AP, AST, ALT, liver and body weight (Additional file 1 Tables S1-S5).

### 3.3. Effect of Zingiber officinale on hepatic antioxidant levels

CCl_4 _group recorded significant decrease in glutathione (63.63%) and total SOD (49.73%) levels, while malondialdehyde showed significant increase by 38.00% (Table 2). Treatment with ethanol extract improved MDA and SOD levels by 60.56 and 10.70%, respectively. Glutathione enhanced after treatment with chloroform and ethanol extracts by 32.70 and 27.97%. Treatment with silymarin recorded improvement in GSH, MDA and SOD by 40.08, 35.21 and 3.84%, respectively.

**Table 2:** Effect of *Zingiber officinale *extracts on hepatic antioxidant levels in CCl_4 _treated rats

Parameters	Control	CCl_4 _group	CCl_4 _treated with ethanol extract	CCl_4 _treated with chloroform extract	CCl_4 _treated with petroleum ether extract	CCl_4 _treated with silymarin
GSH	783.81 ± 124.87 (a)	285.00 ± 59.42 (c)	504.26 ± 59.49 (b)	541.35 ± 85.34 (b)	523.58 ± 66.47 (b)	599.16 ± 128.39 (b)
		
MDA	0.71 ± 0.12 (b)	0.98 ± 0.25 (a)	0. 77 ± 0.04 (b)	0.76 ± 0.12 (b)	0.78 ± 0.14 (b)	0.73 ± 0.03 (b)
		
Total SOD	16.91 ± 2.63 (a)	8.50 ± 1.76 (b)	10.31 ± 2.53 (b)	9.76 ± 2.45 (b)	9.41 ± 1.97 (b)	9.15 ± 2.70 (b)

● Data are means ± SD of eight rats in each group.

● Data are expressed as μg/mg protein for GSH and μmol/mg protein for MDA and total SOD.

● Unshared letters between groups are the significance values at p < 0.0001.

● Statistical analysis is carried out using one way analysis of variance (ANOVA), CoStat Computer Program.

### 3.4. Efficiency of Zingiber officinale on hepatic marker enzymes

Fibrotic liver induced by CCl_4 _recorded significant decrease in SDH, LDH, G-6-Pase, AP and 5'NT by 22.65, 32.50, 24.40, 35.13 and 47.22%, respectively (Table 3). Treatment with ginger extracts recorded improvement in all marker enzymes, whereas ethanolic extract showed higher improvement percentages than the other ones. It recorded enhancement by 13.85, 9.42, 20.60 and 25.41% for SDH, G-6-Pase, AP and 5'NT, respectively. Contradictory, LDH enzyme recorded higher improvement level after treatment with petroleum ether extract (29.9%) (Table 3). Treatment with silymarin improved SDH, LDH, G-6-Pase, AP and 5'NT by 6.39, 23.76, 8.69, 8.18 and 17.77%, respectively.

**Table 3:** Effect of *Zingiber officinale *extracts on hepatic marker enzymes in CCl_4 _treated rats

Parameters	Control	CCl_4 _group	CCl_4 _treated with ethanol extract ethanol extract	CCl_4 _treated with chloroform extract chloroform extract	CCl_4 _treated with petroleum ether extract	CCl_4 _treated with silymarin
SDH	108.86 ± 24.2 (a)	84.2 ± 24.2 (b)	99.28 ± 10.05 (ab)	93.79 ± 6.79 (ab)	96.16 ± 23.51 (ab)	91.16 ± 8.29 (ab)
		
LDH	143.86 ± 18.12 (a)	97.01 ± 41.09 (c)	124.04 ± 27.63 (abc)	100.83 ± 12.93 (bc)	140.06 ± 2.44 (a)	131.2 ± 35.16 (ab)
		
G-6-Pase	43.48 ± 4.83 (a)	32.87 ± 2.24 (b)	36.97 ± 10.31 (ab)	34.07 ± 7.9 (b)	36.55 ± 2.39 (ab)	36.65 ± 6.33 (ab)
		
AP	63.53 ± 6.73 (a)	41.21 ± 2.55 (d)	54.3 ± 3.01 (b)	48.25 ± 3.14 (c)	48.35 ± 4.79 (c)	46.41 ± 3.19 (c)
		
5'NT	304.25 ± 58.81 (a)	160.83 ± 29.38 (d)	238.17 ± 5.79 (b)	215.63 ± 21.42 (bc)	192.18 ± 14.9 (cd)	214.9 ± 47.4 (bc)

● Data are means ± SD of eight rats in each group.

● Data are expressed as μmole/min/mg protein.

● Unshared letters between groups are the significance values at p < 0.0001.

● Statistical analysis is carried out using one way analysis of variance (ANOVA), CoStat Computer Program.

### 3.5. Potency of Zingiber officinale in improving liver function enzymes and serum protein

CCl_4 _group, showed significant increase in AST and ALT levels by 88.34 and 43.52%, respectively (Table 4). However, serum total protein was insignificantly increased by 8.93, 6.97 and 3.91% after treatment with ethanol, chloroform and petroleum ether extracts, respectively. The observed changes in liver function enzymes showed that ethanol extract recorded the most improvement percentages than the other extracts. AST and ALT were ameliorated by 31.11 and 21.26%, while serum protein was improved by 15.19, 13.24 and 12.39% for the three extracts, respectively. Silymarin enhanced AST, ALT and total protein levels by 36.66, 22.84 and 12.39%, respectively.

**Table 4:** Effect of *Zingiber officinale *extracts on liver function enzymes and total protein level in serum of CCl_4 _treated rats

Parameters	Control	CCl_4 _group	CCl_4 _treated with ethanol extract	CCl_4 _treated with chloroform extract chloroform extract	CCl_4 _treated with petroleum ether extract	CCl_4 _treated with silymarin
AST	16.39 ± 1.67 (c)	30.87 ± 3.35 (a)	25.44 ± 2.00 (b)	27.22 ± 2.23 (b)	27.72 ± 2.82 (b)	24.86 ± 3.37 (b)
		
ALT	33.57 ± 4.07 (c)	48.31 ± 6.20 (a)	41.17 ± 8.38 (ab)	42.26 ± 7.45 (ab)	44.82 ± 2.84 (ab)	40.64 ± 7.72 (bc)
		
Serum protein	15.33 ± 4.04 (b)	18.36 ± 1.35 (a)	16.03 ± 0.74 (ab)	16.33 ± 0.72 (ab)	16.46 ± 0.30 (ab)	16.46 ± 1.30 (ab)

● Data are means ± SD of eight rats in each group.

● Liver functions are expressed as Unit/L. Total protein is expressed as mg P/ml.

● Unshared letters between groups are the significance values at p < 0.0001.

● Statistical analysis is carried out using one way analysis of variance (ANOVA), CoStat Computer Program.

### 3.6. Efficacy of Zingiber officinale on serum cholestatic markers

As compared to normal healthy rats, CCl_4 _group recorded significant increase in GGT, ALP and total bilirubin by 55.70, 46.61 and 157.14%, respectively (Table 5). Improvement in serum cholestatic biomarkers after treatment revealed that all extracts shared in improvement by high percentages. Treatment with ethanol extract recorded the highest improving percent in GGT and total bilirubin (53.99 and 154.76%). ALP enhanced by 33.81, 44.74 and 41.29% after treatment with ethanol, chloroform and petroleum ether extracts, respectively. GGT, ALP and total bilirubin recorded improvement percentages reached to 43.85, 33.81 and 152.38% after silymarin treatment.

**Table 5:** Effect of *Zingiber officinale *extracts on serum cholestatic markers in CCl_4 _treated rats

Parameters	Control	CCl_4 _group	CCl_4 _treated with ethanol extract	CCl_4 _treated with chloroform extract	CCl_4 _treated with petroleum ether extract	CCl_4 _treated with silymarin
GGT	15.78 ± 1.53 (bc)	24.57 ± 3.48 (a)	16.05 ± 2.49 (c)	20.25 ± 6.22 (ab)	17.55 ± 4.57 (bc)	17.65 ± 5.46 (bc)
		
ALP	13.90 ± 1.41 (b)	20.38 ± 4.37 (a)	15.68 ± 5.82 (b)	14.16 ± 3.67 (b)	14.64 ± 2.30 (b)	15.68 ± 2.37 (b)
		
Total bilirubin	0.42 ± 0.10 (a)	1.08 ± 1.43 (b)	0.43 ± 0.09 (a)	0.45 ± 0.11 (a)	0.46 ± 0.11 (a)	0.44 ± 0.07 (a)

● Data are means ± SD of eight rats in each group.

● Data are expressed as Unit/L in GGT, ALP and mg/dL in total bilirubin.

● Unshared letters between groups are the significance values at p < 0.001.

● Statistical analysis is carried out using one way analysis of variance (ANOVA), CoStat Computer Program.

### 3.7. Potency of Zingiber officinale in improving liver and relative body weights

CCl_4 _intoxicated rats showed significant increase in liver weight (LW) and its ratio over body weight (LW/BW) by 21.21 and 69.39%, respectively. However body weight (BW) recorded significant decrease by 28.37% (Table 6). Regarding to the recorded changes in LW, BW and their ratios, ethanol extract showed improvement by 17.92, 12.88 and 39.89%, respectively. Treatment with petroleum ether extract showed enhancement by 14.80, 10.66 and 34.42%, while chloroform extract recorded enhancement values by 7.07, 3.92 and 34.69% (Table 6). Silymarin treatment improved LW, BW and their ratio by 0.16, 25.55 and 43.16%, respectively.

**Table 6:** Effect of *Zingber officinale *extracts on liver and body weights of CCl_4 _treated rats

Parameters	Control	CCl_4 _group	CCl_4 _treated with ethanol extract	CCl_4 _treated with chloroform extract	CCl_4 _treated with petroleum ether extract	CCl_4 _treated with silymarin
Liver weight (LW)	6.08 ± 0.71 (b)	7.37 ± 0.95 (a)	6.28 ± 0.59 (b)	6.94 ± 1.06 (ab)	6.47 ± 0.93 (ab)	7.36 ± 0.48 (a)
		
Body weight (BW)	165.66 ± 12.95 (a)	118.66 ± 4.32 (c)	140.00 ± 11.83 (b)	125.16 ± 12.25 (bc)	136.33 ± 26.88 (b)	161.00 ± 6.38 (a)
		
% (LW/BW)	3.66 ± 0.24 (c)	6.20 ± 0.66 (a)	4.74 ± 0.96 (b)	4.93 ± 0.52 (b)	4.94 ± 0.57 (b)	4.62 ± 1.02 (b)

● Data are means ± SD of eight rats in each group.

● Data are expressed as grams.

● Unshared letters between groups are the significance values at p < 0.0001.

● Statistical analysis is carried out using one way analysis of variance (ANOVA), CoStat Computer Program.

### 3.8. Effect of Zingiber officinale on collagen content in CCl_4 _treated liver

**Figure 1:**
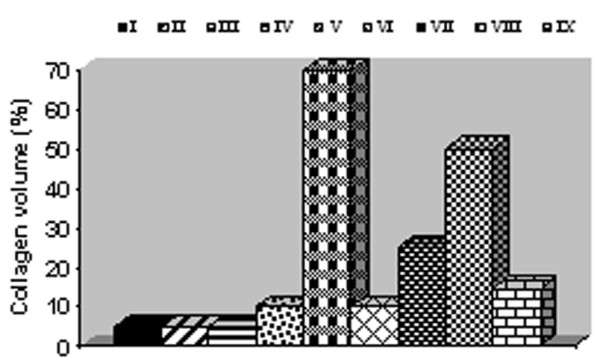
**Percentages of collagen deposition in normal and CCl_4 _treated livers**. Normal control (I), control treated with ethanol extract (II), control treated with chloroform extract (III), control treated with petroleum ether extract (IV), CCl_4 _group(V), CCl_4 _treated with ethanol extract (VI), CCl_4 _treated with chloroform extract (VII), CCl_4 _treated with petroleum ether extract (VIII) and CCl_4 _treated with silymarin (IX).

Normal healthy liver of control and control treated with ethanol and chloroform extracts of ginger recorded 5% collagen deposition. Normal liver treated with petroleum ether extract exerted 10% collagen content. CCl_4 _injured liver revealed 70% collagen deposition. Treatment with ethanol, chloroform, petroleum ether extracts and silymarin recorded decrease in collagen content by 85.71, 64.28, 28.57 and 78.57%, respectively (Figure 1).

### 3.9. Effect of Zingber officinale on liver morphology

**Figure 2:**
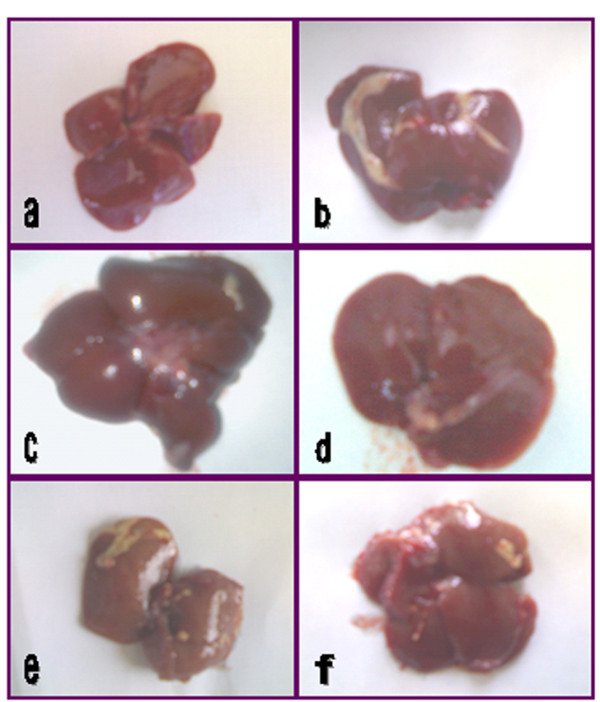
**Morphological structure of normal and CCl_4 _treated livers**. Normal control (a), CCl_4 _injured liver (b), CCl_4 _treated with ethanol extract (c), CCl_4 _treated with chloroform extract (d), CCl_4 _treated with petroleum ether extract (e) and CCl_4 _treated with silymarin drug (f).

CCl_4 _induced changes in liver morphology as compared with normal healthy one (Figure 2-a). Liver colour had changed from normal reddish brown to more or less light brown; a stage of steatosis. The liver enlarges in size and lost it's smoothly surface. Scattered patches of inflammations and pale yellowish fibrotic areas had been seen along with long fibrotic girdle surrounded the outer liver margin (Figure 2-b). Treatment with ethanol extract had returned the liver to it's smoothly appearance, normalized its colour and size and diminished the fibrotic spots to be minimal (Figure 2-c). Chloroform extract of ginger improved the morphological structure of the liver, while a fibrotic area was seen in the hepatic core (Figure 2-d). Petroleum ether extract showed the worst appearance, where large patches as well as many spots of fibrosis were scattered along the liver (Figure 2-e). Treatment with silymarin minimally improved the morphological structure of the liver. Many area of fibrosis along the whole liver surface were spread and marked inflammations along the border of the hepatic lobes were observed (Figure 2-f).

### 3.10. Liver histopathological analysis

**Figure 3:**
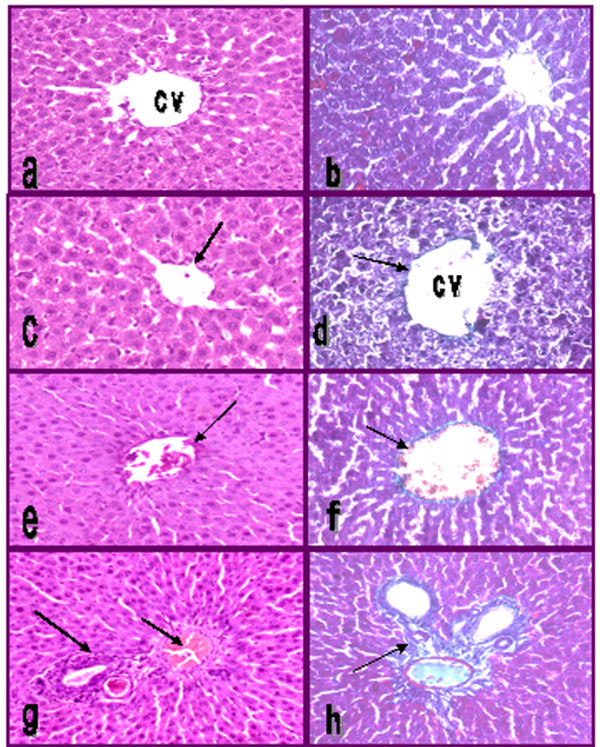
**Photomicrograph of H&E and Masson's trichrom stained liver sections (100×) of control (a,b), control treated with ethanol extract (c,d), control treated with chloroform extract (e,f) and control treated with petroleum ether extract (g,h)**. Central vein (CV). Arrows indicate collagen deposition.

Fig. (3 a, b) showed liver section of control healthy rats with normal hepatic lobular architecture. The hepatocytes were within normal limits and arranged in thin plates. The hepatocytes were separated by narrow blood sinusoids lined by endothelial cells. Portal tracts extend with no fibrous tissue or lymphocytes deposition. Healthy rats treated with ethanolic extract showed normal hepatic lobular architecture. The hepatocytes were within normal limits and no hydropic or steatosis changes. Portal tracts were normal and no sign of fibrosis (Figure 3 c, d). Treatment with chloroform extract of ginger recorded more or less normal hepatocytes architecture (Figure 3 e, f). Petroleum ether extract caused mild hepatocytes aggregation and lymphocytes infiltrations (Figure 3 g, h).

**Figure 4:**
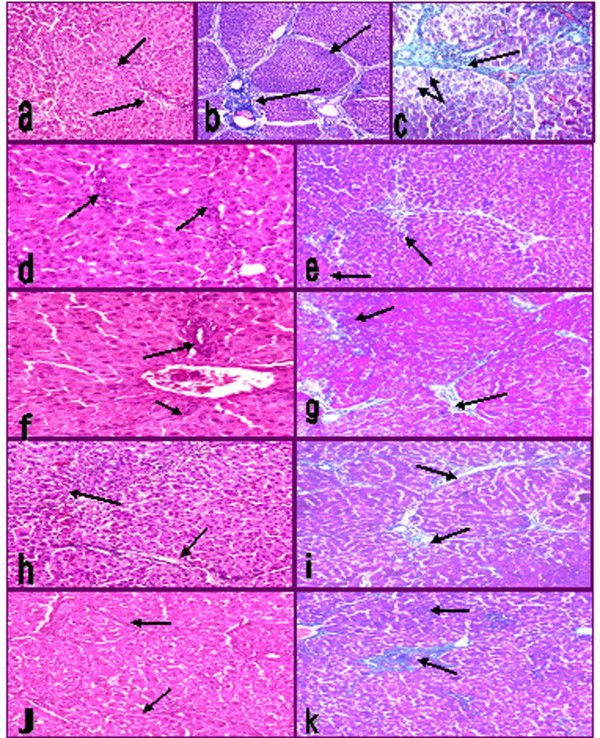
**Photomicrograph of H&E and Masson's trichrom liver sections (100×) of CCl_4 _intoxicated rats (a,b,c), CCl_4 _treated with ethanol extract (d,e) and CCl_4 _treated with chloroform extract (f,g), CCl_4 _treated with petroleum ether extract (h,i), CCl_4 _treated silymarin drug (j,k)**. Arrows indicate collagen deposition and lymphocytes infiltrations. Small arrows indicate fat vacuoles deposition and hepatocytes foamy appearance.

Injured liver with CCl_4 _showed portal loss of hepatic lobular architecture. Ballooning of hepatocytes, deformed cord arrangement and disturbed sinusoids were seen. The hepatocytes showed marked degree of hydropic, steatotic changes and massive necrosis. Portal tracts were extended with marked numbers of chronic inflammatory cells and fibrous tissue. There were porto-portal and porto-central fibrosis. Fibrosis was markedly presented by 70% (Figure 4 a, b, c).

Treatments of injured liver with the ethanol extract showed well formed nucleated hepatocytes arranged in cord with obvious sinusoidal arrays. Minimal fat vacuoles and minimal inflammatory lymphocyte infiltrations were observed. Fibrosis reached to 10% (Figure 4 d, e). Chloroform extract partly preserved the hepatic normal architecture. Mild degrees of steatosis and hydropic changes were recorded. The hepatocytes were still swollen with narrow sinusoids. Portal tracts were extended with moderate fibrous tissue reached to 25% (Figures 4 f, g). Petroleum ether extract showed narrow sinusoids, ballooning and foamy appearance of heapatocytes. Portal tracts were extended with moderate fibrous tissue reached to 50% (Figure 4 h, i). CCl_4 _group treated with silymarin showed well arranged cord of nucleated hepatocytes and sinusoids, while mild fibrotic tissue was still present (15%) (Figure 4 j, k).

## 4. Discussion

Hepatoprotective studies showed that plants have active ingredients that are capable of free radical scavenging in living systems [10]. Flavonoids, sterols, triterpenes and alkaloids as antioxidative compounds are rich in most plants [40]. In the present study and in accordance with Anosike et al. [41], high content of these compounds was recorded in the ethanol extract of ginger followed by chloroform one suggesting their potential role as antioxidative agents.

CCl_4 _is one of an extensively environmental toxicant. The reactive metabolite trichloromethyl radical (CCl_3_^-^) has been formed from the metabolic conversion of CCl_4 _by cytochrome P-450. This reactive metabolite initiates the peroxidation of membrane poly-unsaturated fatty acids (PUFA), generates PUFA radicals, covalently binds to membrane lipids and proteins and generates ROS [42]. Evidence suggests that various enzymatic and non-enzymatic systems have been developed by the cell to attenuate ROS. However, when a condition of oxidative stress establishes, the defense capacities against ROS becomes insufficient. Therefore, ROS affects the antioxidant defense mechanisms, reduces the intracellular concentration of GSH, decreases the activity of SOD and enhances lipid peroxidation [43]. In agreement with these explanations, the observed decrease in SOD was recorded in CCl_4 _treated rats that may be due to inactivation of the antioxidative enzymes. This may cause an increased accumulation of superoxide radicals, which could further stimulate lipid peroxidation. Decrease in GSH activity might be due to decrease availability of GSH resulted during the enhanced lipid peroxidation processes.

Treatment with ginger extracts normalized the antioxidant levels through their rich of flavonoids that have the ability to scavenge free radicals. Silymarin as an antioxidant flavonoid complex derived from the herb milk thistle (*Silybum marianum*), has the ability to attenuate free radicals elevation, chelates metal ions, inhibits lipid peroxidation and prevents liver glutathione depletion [44].

ALT and AST had been reported to be sensitive indicators of liver injury [45]. Significant elevation of ALT and AST after CCl_4 _intoxication was in agreement with those reported studies of Opoku et al. [40] and Gowri Shankar et al. [42]. In addition, Romero et al. [46] showed that CCl_4 _intoxication induced changes in the process of protein synthesis. Hence, increase in total protein content can be deemed as a useful index of the severity of cellular dysfunction in liver diseases as clearly shown in our studies. Stimulation of protein synthesis has been advanced as a contributory self healing mechanism, which accelerates liver regeneration process [47].

Enhancement of cholestatic biomarkers; ALP, GGT and bilirubin were observed in the present study. This was in agreement with Reyes-Gordillo et al. [48] who recorded significant increase in cholestasis biomarker after intoxication of rats with CCl_4_. Leonard et al. [49] confirmed GGT and bilirubin as indicators of bile duct lesions. Hamed [50] added that GGT alone is a poor indicator of cytotoxicity and suggested the combination of other markers like AST, ALT and LDH for accurate detection and early diagnosis. Opoku et al. [40] and Gowri Shankar et al. [42] explained serum enzymes elevation to the increase in hepatic cell membrane fluidity that led to enzyme release into circulation.

Treatment with ginger extracts attenuated the increased level of serum enzymes and caused a subsequent recovery towards normalization. This give an additional support that ginger extracts are able to condition the hepatocytes, accelerate regeneration of parenchyma cells, protect against membrane fragility and decrease leakage of the enzymes into circulation. Therefore, plant extracts acted by the same mode of action of silymarin [42].

Stressful condition also contribute to the oxidative inactivation of G-6-Pase and 5'- nucleotidase [51]. Inhibition of G-6-Pase and 5'NT activities, in our study, are in agreement with the findings of Opoku et al. [40] and Nkosi et al. [52]. The decreased activities of these marker enzymes during CC1_4 _poisoning confirm microsomal and plasma membrane damage. Though these two enzymes are active in two different metabolic pathways, their functional integrity depends on the chemical composition and physical status of the lipid environment where they are embedded. The decrease in membrane phospholipids due to an increase in phospholipase A2 and C and increased lipid peroxidation could be the reason for the decreased enzyme activities [53]. The observed decrease in SDH after CCl_4 _intoxication was in parallel with the finding of Rusu et al. [54]. This decrease in enzyme activity was attributed to the increase in free radicals that affected the inner mitochondrial membrane and intracellular calcium stores led to structural and functional disorganization, over all loss in energy production, irreversible damage, and loss of enzymatic activity [55,56]. Rusu et al. [54] and De-Andrade Belo et al. [57] confirmed the decrease in hepatic LDH after CCl_4 _intoxication. Enzyme inhibition was mainly due to increase membrane fluidity as a result of ROS involvement, leads to enzyme leakage into circulation [50]. The decrease in acid phosphatase enzyme activity was in agreement with Kataria and Singh [58] who observed the same alteration in AP after CCl_4 _induction. Achliya et al. [59] mentioned that the high level of serum AP indicated its inhibition in hepatocytes. The same authors explained the repair mechanisms after treatment to the rise of phospholipids that coupled with thymidylate synthetase and thymidine kinase which confirmed liver regeneration.

Regulatory effect of ginger extracts and silymarin on hepatic marker enzymes was documented in our study as free radicals scavengers [60] that could in turn normalize microsomes, lysosomes, mitochondria and plasma membranes permeability and integrity which lead to restore the hepatic enzymes to its normal levels.

The most commonly associated characteristic of liver fibrosis is the increased deposition of collagens. During liver fibrosis, altered collagen synthesis at both mRNA and protein levels is observed, with a dramatic increase in type I collagen along with smaller, but significant, increases in type III collagen. This increase in collagen deposition gave an additional support of the observed increase in liver weight and its ratio over body weight. The excess deposition of extracellular matrix proteins disrupts the normal architecture of the liver, alters its normal function and ultimately leading to pathophysiological damage [61]. The most remarkable pathological characteristics of CCl_4 _-induced hepatotoxicity are massive centrilobular necrosis, ballooning degeneration, cellular infiltration and steatosis [42]. This was in accordance with the present finding of massive deformation of hepatic cells architecture. In ginger and silymarin treated groups, hepatocyte degeneration, necrosis and infiltration of inflammatory cells were all apparently ameliorated. Collagen deposition was markedly reduced. Minimal deposition of fat vacuoles was apparently observed. Silymarin has anti-fibrotic and anti inflammatory effects, inhibit activation of hepatic stellate cells through the expression of transforming growth factor-beta1 and stabilization of mast cells [62]. We compare the anti-fibrogenic effects of silymarin with extracts and the results exhibited that ethanol extract had higher potency in inhibiting collagen deposition and fibrosis severity.

## Conclusions

Ginger has the ability to down regulate free radicals elevation, improve liver and cholestatic biomarkers, ameliorate hepatic marker enzymes, reduce collagen deposition, fibrosis severity and normalize the hepatic cells architecture. Ginger ethanolic extract recorded the most potent effect in improving the selected parameters. The hepatoprotective effect of *Z. officinale *demonstrated in this study may explore its nutraceutical role in human diet. Therefore, further studies are need for its clinical application.

## Competing interests

The authors declare that they have no competing interests.

## Authors' contributions

TKM, RMH: Participated in the experimental design and final form of manuscript revision. MAH: Design the experiment, participated in the biochemical analysis and data collections. Performed the statistical analysis, drafted and revised the manuscript. MHS: Plant extractions and phytochemical screening test. AFA: Carried out the experiments and participated in data collections under supervision of MAH. All authors read and approved the final manuscript.

## Supplementary Material

Additional file 1 **Effect of successive extracts of *Zingiber officinale *on certain biochemical parameters in normal control rats**. The data provided represent the effect of successive ginger extracts on hepatic antioxidant levels, hepatic marker enzymes, liver function tests, cholestatic indices and liver and body weights in normal healthy rats Click here for file
