# Supplementary material for: Zingiber officinale acts as a nutraceutical agent against liver fibrosis
Source: Nutr Metab (Lond). 2011 Jun 20;8:40. doi: 10.1186/1743-7075-8-40 (PMC3199745; doi:10.1186/1743-7075-8-40)
Supplement: Additional file 1 — Effect of successive extracts of Zingiber officinale on certain biochemical parameters in normal control rats. The data provided represent the effect of successive ginger extracts on hepatic antioxidant levels, hepatic marker enzymes, liver function tests, cholestatic indices and liver and body weights in normal healthy rats [file 1743-7075-8-40-S1.DOC]

Table S1: Effect of *Zingiber officinale* extracts on hepatic antioxidant levels in normal healthy rats.

| Parameters | Control | Control treated with ethanol extract | Control treated with chloroform extract | Control treated with petroleum ether extract |
| --- | --- | --- | --- | --- |
|
| GSH | 740.65±66.23  (a) | 738.38±44.40  (a) | 723.96±55.32  (a) | 675.00±56.65  (a) |
| MDA | 0.71±0.21  (a) | 0.72±0.03  (a) | 0.73±0.07  (a) | 0.77±0.08  (a) |
| Total SOD | 16.91±2.63  (a) | 15.23±1.96  (a) | 16.08±2.17  (a) | 15.06±2.74  (a) |

- Data are means ± SD of eight rats in each group.
- Data are expressed as µg/mg protein for glutathione and µmol/mg protein for MDA and total SOD.
- Shared letters between groups are insignificant values.
- Statistical analysis is carried out using one way analysis of variance (ANOVA), Co Stat Computer Program.

Table S2: Effect of *Zingiber officinale* extracts on hepatic marker enzymes in normal healthy rats.

| Parameters | Control | Control treated with ethanol extract | Control treated with chloroform extract | Control treated with petroleum ether extract |
| --- | --- | --- | --- | --- |
|
| SDH | 108.86±24.2  (a) | 108.16±14.91  (a) | 96.2±11.88  (a) | 93.99±9.47  (a) |
| LDH | 143.86±18.12  (a) | 128.13±5.49  (a) | 141.45±6.31  (a) | 128.89±22.16  (a) |
| G-6-Pase | 43.48±4.83  (a) | 40.63±4.80  (a) | 40.60±9.86  (a) | 42.23±2.82  (a) |
| AP | 56.93±5.93  (a) | 54.55±2.06  (a) | 56.55±2.15  (a) | 43.52±6.18  (b) |
| 5`NT | 270.60±39.95  (a) | 257.24±2.36  (a) | 253.43±5.21  (a) | 265.15±11.54  (a) |

- Data are means ± SD of eight rats in each group.
- Data are expressed as µmole /min/ mg protein.
- Unshared letters between groups are the significance values at p< 0.0001.
- Statistical analysis is carried out using one way analysis of variance (ANOVA), CoStat Computer Program.

Table S3: Effect of *Zingiber officinale* extracts on liver function enzymes and protein level in serum of normal healthy rats.

| Parameters | Control | Control treated with ethanol extract | Control treated with chloroform extract | Control treated with petroleum ether extract |
| --- | --- | --- | --- | --- |
|
| AST | 16.39±1.67  (b) | 18.22±1.43  (b) | 18.29±2.44  (b) | 22.55±1.17  (a) |
| ALT | 33.57±4.02  (b) | 34.66±9.31  (b) | 37.24±3.19  (b) | 48.56±7.34  (a) |
| Serum protein | 15.33±4.02  (a) | 16.70±0.89  (a) | 16.40±0.70  (a) | 15.93±0.39  (a) |

- Data are means ± SD of eight rats in each group.
- Data are expressed as U/L and serum protein as mg/ml.
- Unshared letters between groups are the significance values at p< 0.0001.
- Statistical analysis is carried out using one way analysis of variance (ANOVA), CoStat Computer Program.

Table S4: Effect of *Zingiber officinale* extracts on serum cholestatic markers in normal healthy rats.

| Parameters | Control | Control treated with ethanol extract | Control treated with chloroform extract | Control treated with petroleum ether extract |
| --- | --- | --- | --- | --- |
|
| GGT | 15.78±1.53  (a) | 18.68±6.83  (a) | 20.48±6.11  (a) | 19.69±3.41  (a) |
| ALP | 13.90±1.41  (a) | 14.55±3.46  (a) | 14.67±2.77  (a) | 15.43±2.54  (a) |
| Total bilirubin | 0.42±0.10  (a) | 0.47±0.09  (a) | 0.47±0.06  (a) | 0.43±0.10  (a) |

- Data are means ± SD of eight rats in each group.
- Data are expressed as Unit/L for GGT, ALP and mg/dL for total bilirubin.
- Shared letters between groups are non significance.
- Statistical analysis is carried out using one way analysis of variance. (ANOVA), CoStat Computer Program.

Table S5: Effect of *Zingiber officinale* extracts on liver and body weights of normal healthy rats.

| Parameters | Control | Control treated with ethanol extract | Control treated with chloroform extract | Control treated with petroleum ether extract |
| --- | --- | --- | --- | --- |
|
| LW | 6.08±0.71  (a) | 5.45±0.45  (a) | 5.63±0.50  (a) | 4.49±0.29  (b) |
| BW | 165.16±12.95  (a) | 153.95±33.50  (ab) | 153.8±15.30  (ab) | 134.38±6.67  (b) |
| % (LW/BW) | 3.63±0.16  (ab) | 3.38±0.34  (b) | 3.50±0.23  (a) | 3.36±0.22  (b) |

- Data are means ± SD of eight rats in each group.
- Data are expressed in grams.
- Unshared letters between groups are the significance values at p< 0.0001.
- Statistical analysis is carried out using one way analysis of variance (ANOVA), CoStat Computer Program.
